# Prevalence of Trachoma in Pre-validation Surveillance Surveys in 11 Evaluation Units (Covering 12 Districts) in Oromia Regional State, Ethiopia: Results from 2018−2020

**DOI:** 10.1080/09286586.2022.2119258

**Published:** 2022-12-15

**Authors:** Hirpa Miecha, Michael Dejene, Dereje Adugna, Ageru Kebede, Damtew Yadeta, Addisu Alemayehu, Aemero Abateneh, Mihiret Dayessa, Muhammed Shafi, Emawayish Taye, Leta Balcha, Nebiyu Negussu, Belete Mengistu, Rebecca Willis, Cristina Jimenez, Ana Bakhtiari, Sarah Boyd, Biruk Kebede, Fantahun Tadesse, Ayele Mamo, Mengistu Bekele, Zelalem Sinke, Anthony W. Solomon, Emma M. Harding-Esch

**Affiliations:** a Oromia Regional State Health Bureau, Addis Ababa, Ethiopia; b Sightsavers, Addis Ababa, Ethiopia; c The Fred Hollows Foundation, Addis Ababa, Ethiopia; d Research Triangle Institute, Addis Ababa, Ethiopia; e Jimma University, Jimma, Ethiopia; f Ambo Hospital Ambo, Ethiopia; g Adama Hospital Medical College, Adama, Ethiopia; h International Trachoma Initiative, Task Force for Global Health, Decatur, GA, USA; i Sightsavers International, Haywards Health, UK; j Department of Control of Neglected Tropical Diseases, World Health Organization, Geneva, Switzerland; k Clinical Research Department, London School of Hygiene & Tropical Medicine, London, UK

**Keywords:** trachoma, trichiasis, epidemiology, recrudescence

## Abstract

**Purpose:**

Interventions to reduce the prevalence of trachoma and transmission of ocular *Chlamydia trachomatis* have been implemented in Oromia Region, Ethiopia. Following an impact survey in which the trachomatous inflammation—follicular (TF) prevalence in 1–9-year-olds is <5%, a surveillance survey is recommended 2 years later, without additional antibiotic treatment. We report results of surveillance surveys in 11 evaluation units (EUs) covering 12 districts in Oromia Region, to plan whether future interventions are needed.

**Method:**

We use a two-stage cluster-sampling cross-sectional survey design. In each EU, 26 clusters (villages) were systematically selected with probability proportional to size; from each cluster, 30 households were selected using compact segment sampling. Water, sanitation and hygiene (WASH) access was assessed in all selected households. All residents of selected households aged ≥1 year were examined for TF and trachomatous trichiasis (TT) by certified graders.

**Result:**

Of 31,991 individuals enumerated, 29,230 (91% of) individuals were examined. Eight EUs had an age-adjusted TF prevalence in 1−9-year-olds of ≥5% and seven had a TT prevalence unknown to the health system among adults aged ≥15 years of ≥0.2%. About one-third of visited households had access to an improved water source for drinking, and 5% had access to an improved latrine.

**Conclusion:**

Despite TF reductions to <5% at impact survey, prevalence recrudesced to ≥5% in all but three of the 11 EUs. Operational research is needed to understand transmission dynamics and epidemiology, in order to optimise elimination strategies in high-transmission settings like these.

## Introduction

Trachoma is an eye disease caused by certain strains of the *Chlamydia trachomatis* bacterium. Infection is frequently first encountered in infancy or childhood and in endemic areas, recurs frequently.^1,2^ Transmission is thought to occur through transfer of eye and nose secretions, directly via contact with hands and faces of infected people, or indirectly via fomites (shared clothing) or the eye-seeking *Musca sorbens* fly.^3,4^ Access to water, sanitation and hygiene (WASH) is considered key to limiting transmission.^5^ Consequently, the World Health Organization (WHO)-recommended strategy for trachoma elimination as a public health problem, known by the acronym “SAFE”, includes facial cleanliness (F) and environmental improvement (E); the S and A components of SAFE are surgery (for trachomatous trichiasis, TT) and antibiotic mass drug administration (MDA; to clear infection), respectively.^6^

Trachoma is a public health problem in 44 countries, and is responsible for the blindness or visual impairment of about 1.9 million people. Based on March 2020 data, 137 million people globally live in trachoma endemic areas and are at risk of trachoma blindness.^7^

Africa is the continent most affected by trachoma. Ethiopia is the second most populous country in Africa and has the highest national burden of trachoma worldwide.^7–9,10^

Oromia Regional State is Ethiopia's most populous region.^11^ Oromia has more than 39 million people an area of about 363,136 km^2^ and is administratively divided into 21 zones and 337 woredas (districts).^12^

Oromia Regional Health Bureau conducted population-based trachoma prevalence surveys between December 2012 and July 2014, using Global Trachoma Mapping Project (GTMP) protocols.^9^ These surveys revealed that 56 evaluation units (EUs) covering 218 districts needed implementation of the A, F and E components of the SAFE strategy for 3 years before impact surveys could be undertaken. It was further noted that 72 EUs covering 240 districts had TT prevalence above the elimination threshold of 0.2% in those aged ≥15 years.^9^

Starting from 2013, SAFE has been implemented in Oromia. About 256 integrated eye care workers (IECWs) have been trained and deployed with the necessary equipment to undertake TT surgery. As a result, over 146,724 TT cases have been operated, compared to the previously estimated backlog of 200,000, contributing to the national TT backlog clearance initiative. In addition, more than 113 million doses of azithromycin (Zithromax, Pfizer Inc, New York, NY, USA; donated through the International Trachoma Initiative, Decatur, GA, USA)^13^ have been distributed in EUs requiring MDA. Much effort has been invested developing clean water sources, constructing latrines and encouraging latrine usage throughout the region by the regional government. In addition, awareness creation activities have been implemented by health extension workers, as part of the regional health sector plan, on the importance of face washing, hand hygiene and environmental sanitation activities to reducing individual risk of contracting trachoma.^12^

In this manuscript, we present trachoma prevalence data from surveillance surveys conducted in 11 EUs (covering 12 districts) of Oromia between 2018 and 2020. These surveys were undertaken, as recommended by WHO,^14^ after impact surveys demonstrated a trachomatous inflammation—follicular (TF) prevalence in 1–9-year-olds of <5%, in order to see whether reductions in TF prevalence had been sustained in the absence of ongoing MDA.

## Methods

### Ethics approval and consent to participate

Ethical approval was obtained from Oromia Regional State Health Bureau ethical clearance committee (protocol no. BEFO/AHDFIDh/1-69/3079). Official letters of permission were also obtained from each concerned zone and district health office. Tropical Data survey support was approved by the London School of Hygiene & Tropical Medicine Observational Ethics Committee (16105).

Before the commencement of data collection, each individual taking part in these surveys was informed about their purpose, procedures and confidentiality of the information provided. Consent was recorded in Android smartphones. Parents or guardians of children gave consent on their children’s behalf. Individuals diagnosed with active trachoma (TF and/or trachomatous inflammation—intense, TI) or any other likely-bacterial conjunctivitis were provided with two tubes of 1% tetracycline eye ointment to be used twice daily for six weeks and shown how to administer it. People identified with TT were referred to the nearest appropriate health facility for management.

### Survey design, participant selection

Community-based cross-sectional surveys were conducted. For the purposes of trachoma elimination, WHO defines an EU as “the normal administrative unit for health-care management, consisting of a population unit between 100,000 and 250,000 persons”.^15^ In these surveys, eleven EUs covering twelve districts from five zones of Oromia were included. A two-stage cluster sampling technique was employed to select study participants. In each EU, lists of clusters (defined for these surveys as a gare, a sub-unit of a village with an average of 30 households each) were obtained from district health offices. Among these lists, 30 survey clusters were randomly selected per EU for data collection. A structured questionnaire on access to WASH facilities was administered to the head (or another adult resident) of each selected household, and all residents of the household aged ≥1 year were invited to have both eyes examined for trachoma.

### Sampling and sample size

Survey sample size was estimated using the single population proportion for precision formula.^16^ We aimed to estimate TF prevalence of 4% in 1–9-year-olds with a precision of ±2% at the 95% confidence level. Our estimated mean number of 1–9-year-old children per household was 1.5, based on the expectation that 1–9-year-olds make up 35% of the total population.^17^ Assuming a design effect of 2.63^18^ and after factoring in a non-response inflation factor of 1.2, it was determined that 1,164 children aged 1–9 years should be enumerated per EU. Twenty-six to twenty-eight clusters comprising 30 households were needed in each EU to enumerate that number of 1–9-year-olds.

For all EUs, the sample for estimation of TT prevalence was composed of adults living in the households selected to survey children. Because TT was expected to be rarer than TF, the TT prevalence estimate will have less relative precision than the TF estimate.

### Training

Using trainers certified to train graders and recorders by Tropical Data, training and certification of survey graders and recorders was performed, followed by combined training of field teams on survey procedures. We used the training system in the second and third versions of the Tropical Data Training manual (please see below for details of version differences).^19,20^

### Data collection

All data were captured electronically using a purpose-built Open Data Kit-based Android Smartphone application. During each day of fieldwork, survey teams moved house-to-house collecting data. Once household heads had agreed that their house could be enrolled, the Global Positioning System (GPS) coordinates of the household were recorded and the recorders administered the WASH questionnaire. WASH questions were derived from the United Nations Children’s Fund (UNICEF)/WHO Joint Monitoring Programme (JMP), modified for trachoma surveys.^21^ In this survey series, due to an update in the data collection form that occurred part-way through the series, two versions of the WASH questionnaire were employed for hand washing facility observation. Version two was used in Gechi, Mulo & Bereh, Sebeta Hawas, Sululta, Welmera, Aleltu, Kimbibit and A/G/Beret. In it, data recorders observed handwash stations within 15 m of the latrine; where there was no latrine, no handwash station observations were carried out. Version three was used in Botor Tolay, Omo Beyam and Omo Nada; data recorders observed handwash stations on the household premises or in the yard outside the premises, regardless of the presence or absence of a latrine. Water source type, water source proximity, latrine ownership and latrine status were the same between the two versions: only the handwash station question differed. Each individual consented for inclusion was examined using binocular 2.5× magnifying loupes, and sunlight or a torch for illumination. Certified graders determined the presence or absence of trichiasis, TF and TI according to the then-current WHO simplified grading system. As a result of recommendations made at the 4th Global Scientific Meeting on Trachoma (GSM4) in November 2018, the definition of TT in the WHO simplified system was refined part way through this survey series (at the same time as the change from version 2 to version 3 of the WASH questionnaire). TT was therefore graded slightly differently in the surveys conducted using the pre-GSM4 methodology (Gechi, Mulo & Bereh, Sebeta Hawas, Sululta, Welmera, Aleltu, Kimbibit and A/G/Beret) compared to the surveys conducted using the post-GSM4 methodology (Botor Tolay, Omo Beyam and Omo Nada). In the former, TT was defined as at least one eyelash touching the eyeball or evidence of recent epilation of in-turned eyelashes,^22^ and the eyelid from which the aberrant eyelash(es) emanated was not recorded. In the latter, TT was defined as at least one eyelash from the upper eyelid touching the eyeball, or evidence of recent epilation of in-turned eyelashes from the upper eyelid.^23^ In all 11 EUs, regardless of whether pre- or post-GSM4, TT grading protocols were used. Where trichiasis was recorded as being present in an eye, the individual was asked whether they had been offered management for it, and whether they had accepted it.^24^ Those who had not been offered surgery or could not remember having been offered it, for at least one eye with TT were considered to have TT “unknown to the health system”.^25^ Where one or more resident 1–9-year-old was missing at the time of the first visit, the household was re-visited once before the end of the day.

### Quality issues

One survey coordinator assigned by the Regional Health Bureau had responsibility for linking survey teams with local authorities, communicating with the regional team in case of any issues in the field, overseeing the survey team, checking availability of necessary resources and logistics on a daily basis, and checking the security status of the targeted EUs before field deployment. In addition, two-layer supervision was used to maintain survey quality. The first layer involved supervisors who were non-ophthalmologist eye health professionals qualified as supervisors and grader trainers (scoring kappas ≥0.8 in field inter-grader agreement tests against Tropical Data reference graders). These first-line supervisors were generally responsible for seven survey teams and traveled with them each day to provide technical support. They oversaw whether the team was properly following the survey protocol or deviating from it, checked how well the team was recording necessary data and occasionally undertook examinations where graders were not able to do so. The second layer was composed of lead supervisors/trainers who were ophthalmologists, providing technical backup to both first-line supervisors and data collectors whenever required. The senior supervisors provided weekly plan to each team, made sure that data were collected from all targeted clusters as per the plan, technically assisted survey team in case they encountered complicated eye health problems that were beyond the capacity of graders/first line supervisors, verified lower eyelid trichiasis cases or TT cases in those aged <15 years whenever they were reported, and reported to regional and national team leads. Quality control and quality assurance measures used throughout the survey process are described in more detail elsewhere.^26,27^

### Data analysis

Data were stored on the smartphone’s micro-secure digital (SD) card, until a data-enabled mobile network or WiFi signal was available then transmitted to a central database for cleaning and analysis. Analysis of TF and TT prevalence, including adjustments for age and gender and estimation of confidence intervals (CIs), was conducted as described elsewhere.^21,28^ WASH facilities were categorized in line with UNICEF/WHO JMP recommendations.

## Results

Fieldwork took place between January 2017 and March 2020. Across 11 EUs, a total of 31,991 individuals were enumerated, 29,230 consenting individuals were examined and 2,528 individuals were absent on the day of the household visit. Fifty-four percent of examined individuals were female. Details from each EU are shown in Table 1.

**Table 1.** Population enumerated and participants examined in 11 trachoma surveillance survey evaluation units (EUs) of Oromia Region, Ethiopia, January 2017–March 2020.

z	Total Enumerated	Total Female Enumerated	Total Examined	Total Female Examined	Total Absent	Total Refused
Gechi	3075	1536	2870	1480	193	12
Mulo & Bereh	2916	1451	2480	1340	386	50
Sebata Awas	2920	1432	2513	1347	349	58
Sululta	2991	1517	2538	1408	410	43
Welmera	2942	1488	2578	1413	331	33
Aleltu	3103	1510	2857	1464	230	16
Kimbibit	3062	1491	2777	1438	268	17
Abuna Gindeberet	2988	1561	2817	1504	169	2
Boter Tolay	2537	1357	2472	1345	64	1
Omo Beyam	2751	1499	2690	1475	61	0
Ommo Nadda	2706	1507	2638	1493	67	1
Total	31991	16349	29230	15707	2528	233

Eight of 11 EUs had an age-adjusted TF prevalence estimate in 1–9-year-olds ≥5%, above the trachoma elimination threshold.^29^ There was considerable variation in TF prevalence between EUs: the lowest (2.7%, 95%CI 2.1–5.4) was reported in Aleltu district, while the highest (13.8%, 95%CI 8.9–20.4) was reported in Sebata Hawas district (Table 2).

**Table 2.** Prevalence of trachomatous inflammation—follicular (TF) in 11 trachoma surveillance survey evaluation units (EUs) of Oromia Region, Ethiopia, January 2017–March 2020.

EU	1–9-year-olds examined	1–9-year-olds with TF	Age-adjusted prevalence of TF in 1–9- year-olds (%)	95% confidence interval around TF prevalence estimate
Gechi	1251	47	3.8	[1.9–6.5]
Mulo & Bereh	876	60	6.7	[3.3–7.8]
Sebeta Hawas	893	123	13.8	[8.9–20.4]
Sululta	976	65	6.6	[3.9–9.7]
Welmera	904	78	8.6	[3.5–10.6]
Aleltu	1060	29	2.7	[2.1–5.5]
Kimbibit	1133	50	4.4	[3.3–7.4]
Abuna Ginda Beret	1089	98	9.0	[6.0–13.3]
Botor Tolay	976	64	6.6	[3.6–9.5]
Omo Beyam	1236	89	7.0	[4.5–12.5]
Omo Nada	1123	120	10.7	[6.5–13.1]
**Total**	**11,517**	**823**		

**Figure 1.**
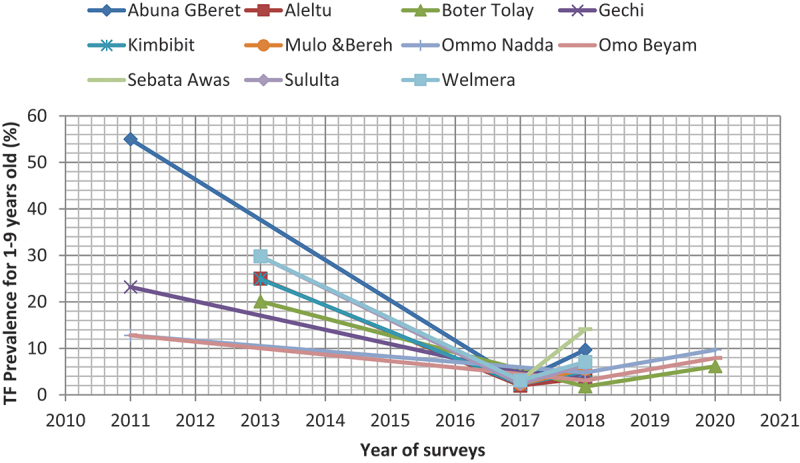
Trachomatous inflammation—follicular (TF) prevalence in 1–9-year-olds over time, 11 evaluation units, Oromia Region, Ethiopia. (Methodologies and results of baseline and impact surveys are published elsewhere.)^30,31^

TF prevalence estimates were lower than at baseline for all surveyed EUs (Figure 1). For example, the TF prevalence in Abuna Gindeberet was 55% at baseline (in 2011); at impact survey (in 2017), following five rounds of MDA, it was 3%, and at the time of surveillance survey (in 2018), it was 9.0%. The TF prevalence was <5% at the time of these surveillance surveys only in Gechi, Kimbibit and Aleltu.

Of 17,713 individuals aged ≥15 years examined, 179 had TT. In 7 of the 11 EUs the age- and gender-adjusted TT prevalence unknown to the health system among ≥15-year-olds was ≥0.2%. Four EUs (Mulo & Bereh, Sululta, Welmera and Kimbibit had a TT prevalence <0.2%, Table 3).

**Table 3.** Prevalence of trachomatous trichiasis (TT) in ≥15-year-olds (%), 11 trachoma surveillance survey evaluation units (EUs), Oromia Region, Ethiopia, January 2017–March 2020.

EU	≥15-year-olds examined	Data from pre-GSM4 surveys (both upper and lower eyelid trichiasis included as “TT”)				Data from post-GSM4 surveys (only upper eyelid trichiasis included as “TT”)			
		≥15-year-olds with TT	≥15-year-olds with TT unknown to the health system	Age- and gender-adjusted prevalence of TT unknown to the health system in ≥15-year- olds (%)	95% confidence interval around age- and gender-adjusted prevalence of TT unknown to the health system in ≥15- year-olds (%)	≥15-year-olds with upper eyelid TT	≥15-year-olds with upper eyelid TT unknown to the health system	Age- and gender-adjusted prevalence of upper eyelid TT unknown to the health system in ≥15-year-olds (%)	95% confidence interval around age- and gender-adjusted prevalence of upper eyelid TT unknown to the health system in ≥15- year-olds (%)
Gechi	1619	11	8	0.32	0.2–0.9	NA	NA	NA	NA
Mulo& Bereh	1604	6	4	0.12	0.0–0.4	NA	NA	NA	NA
Sebeta Hawas	1620	21	13	0.49	0.4–1.1	NA	NA	NA	NA
Sululta	1562	8	6	0.19	0.0–0.5	NA	NA	NA	NA
Welmera	1674	8	4	0.09	0.0–0.7	NA	NA	NA	NA
Aleltu	1797	12	11	0.62	0.2–1.4	NA	NA	NA	NA
Kimbibit	1644	5	3	0.11	0.0–0.3	NA	NA	NA	NA
A/G/Beret	1728	29	18	0.99	0.5–2.2	NA	NA	NA	NA
Botor Tolay	1496	NA	NA	NA	NA	13	11	0.61	0.2–1.4
Omo Beyam	1454	NA	NA	NA	NA	17	16	0.64	0.3–1.1
Omo Nada	1515	NA	NA	NA	NA	49	39	1.27	1–1.9
**Total**	**17,713**	**100**	**67**			**79**	**66**		

Overall, about a quarter (23%) of 8,804 visited households from the 11 EUs reported access to an improved source of water for drinking within a 30-minute return journey. The proportion of households which reported access to an improved source of water for drinking within a 30-minute journey ranged between 20% in Botor Tolay district and 39% in Mulo and Bereh districts. Only 5% of all visited households had access to an improved latrine. Household access to improved latrines ranged from 0% in Aleltu to 21% in Abuna Ginde Beret. In all EUs, the proportion of households with a hand washing station with water and soap was <5% (Table 4).

**Table 4.** Household-level access to water, sanitation and hygiene facilities, trachoma surveillance surveys, Oromia Region, Ethiopia, January 2017–March 2020.

Evaluation unit	Number of clusters	Number of house holds	Households with an improved drinking water source within 30- minute journey (number [%])	Households with an improved latrine (number [%])	Households with a hand wash station with water & soap within 15 m of latrine (number [%])	Households with a hand wash station with soap and water on the household premises (number [%])
Gechi	26	789	276 [35]	1 [0.1]	1 [0.1]	NA
Mulo& Bereh	28	836	166 [20]	8 [1.0]	3 [0.4]	NA
Sebta Haws	28	840	194 [23]	15 [1.8]	12 [1.4]	NA
Sululta	26	798	224 [28]	59 [7.4]	14 [1.8]	NA
Welmera	28	846	308 [36]	5 [0.6]	15 [1.8]	NA
Aleltu	26	789	221 [28]	0 [0.0]	1 [0.1]	NA
Kimbibit	26	791	286 [36]	11[1.4]	3 [0.4]	NA
A/G/Beret	26	779	196 [25]	163 [20.9]	38 [4.9]	NA
Botor Tolay	26	779	304 [39]	83 [10.7]	NA	26 [3.3]
Omo Beya	26	780	225 [29]	39 [5.0]	NA	1 [0.1]
Omo Nada	26	777	238 [31]	48 [6.2]	NA	1 [0.1]
**Total**		**8,804**	**2,638 [**23]	**432 [**4.9]	**87**	**28**

## Discussion

According to WHO recommendations, EUs with a TF prevalence in 1−9-year-olds <5% at impact survey should receive no further MDA for 2 years and then be re-surveyed to determine whether the low TF prevalence has been sustained.^14^ At impact survey, all 11 EUs described in this manuscript had TF prevalence <5%.^30^ However, by the time of the surveillance surveys reported here, two years after MDA was stopped, only three EUs (Aleltu, Kimbibit and Gechi) had maintained TF <5%. The prevalence of TF was in the 5.0−9.9% range in six EUs, and >10-29.9% in two EUs. One or three renewed rounds of MDA respectively are now warranted in these EUs to further reduce the prevalence of *C. trachomatis* infection.^32^

Our surveys cannot provide explanations as to why TF recrudesced in these EUs, but we can speculate. It is notable, for example, that despite ongoing work, the overall coverage of WASH access in these EUs was low. More than three-quarters of households surveyed had to undertake a round trip of >30 minutes to reach an improved drinking water source. Living in water-scarce environments such as these may discourage face washing.^33,34^ Similarly, more than 95% of households did not access an improved latrine, suggesting an abundance of breeding sites for eye-seeking flies in these communities.^35^ A reduction in improved latrine coverage was observed in four of the seven EUs for which data were available for both the baseline and surveillance surveys. These metrics are suggestive of an environment at high risk of ocular *C. trachomatis* transmission, and are associated with high TF prevalence.

The adjusted prevalence of TT unknown to the health system in ≥15-year-olds remained above the elimination target in seven of the 11 surveyed EUs. Only four had achieved a prevalence of <0.2%. This is disappointing given the efforts that have been made to provide TT surgical services in the region. Surgical services should be further strengthened and active case finding undertaken throughout the region to identify and manage cases of TT. TT prevalence estimates should also be interpreted with caution as the sample size was powered to provide precision around the TF prevalence estimate. According to WHO recommendations, either 2818 adults aged ≥ 15 years should be examined, or 30 clusters per EU surveyed^36^: neither of these conditions were met in any of these surveys

We acknowledge additional limitations to our work. First, because of differences in sampling frame and methodology, comparisons with data before this study should be made with extreme caution. In the baseline surveys covering the EUs described here, multiple districts were surveyed as a single EU.^25^ In impact and surveillance surveys, those EUs were re-framed with smaller population sizes (single districts for most EUs; two districts for one EU). Where multiple districts were surveyed as a single EU at baseline, baseline prevalences of the parent EU were assigned to each of the individual offspring EUs for the purposes of Figure 1, though this is not really epidemiologically valid. Pre-MDA estimates generated using the Ethiopia National Trachoma Programme protocol^31^ had even greater methodological differences to these surveillance surveys than did the pre-MDA estimates generated with support from the GTMP. It is therefore difficult to contextualize the findings from this study using baseline and impact survey data. Second, we collected data on WASH infrastructure, but not on objective WASH facility usage or other behaviors^37,38^ which might be relevant to transmission of infection. It is likely that important drivers of transmission went unmeasured here and we continue to be unaware of their potential impact on prevalence. Though the evidence base for the F and E components of SAFE is weak,^39,40^ modeling suggests that small differences in transmission intensity could make critical differences to trachoma elimination prospects over the longer term.^41^

The progress of Oromia, the largest region of Ethiopia, towards elimination of trachoma as a public health problem is of paramount importance to the global trachoma elimination programme. It is disappointing that these surveillance surveys indicate that TF prevalence targets were not maintained two years after MDA cessation. With these data, however, we can plan for further interventions and operational research to improve future strategies; that work has already begun.^42–46^ It is also clear that improvements in WASH are needed, not just for trachoma elimination purposes or for the broader neglected tropical disease agenda, but to achieve general improvement in the quality of life of people living in this region.^47^
